# Transrectal focal HIFU: the use of MRI fusion in guiding treatment

**DOI:** 10.1186/2050-5736-3-S1-O57

**Published:** 2015-06-30

**Authors:** Stephen Scionti

**Affiliations:** 1 Scionti Prostate Center, Sarasota, Florida, United States

## Background/introduction

The ability to effectively ablate prostate cancer with transrectal HIFU has been demonstrated by numerous publications to date. Early results from focal HIFU series suggest that side effect profiles such as stricture, erectile dysfunction, and incontinence can be greatly reduced as compared to total gland ablative HIFU treatment.

## Methods

Multiparametric MRI combined with both systematic and fusion biopsy is being utilized more frequently worldwide to select patients for focal therapy.

The ability to effectively ablate prostate cancer with transrectal HIFU has been demonstrated by numerous publications to date. Early results from focal HIFU series suggest that side effect profiles such as stricture, erectile dysfunction, and incontinence can be greatly reduced as compared to total gland ablative HIFU treatment. Multiparametric MRI combined with both systematic and fusion biopsy is being utilized more frequently worldwide to select patients for focal therapy. Commercially available, FDA approved MRI to ultrasound fusion platforms are now readily available for use in the urology clinic setting, however, these have not yet been widely used to guide focal HIFU treatment. The multistep workflow requires:

1. Creation of a 3D MRI model using MRI images of the prostate that contain a lesion proven by targeted biopsy to be malignant;

2. Creation of a 3D model of the prostate using ultrasound images, rigid and elastic image fusion; and

3. Use of the fused images to guide treatment.

This process will be demonstrated using clinical examples and progress on the integration of an MRI to ultrasound fusion system into the Sonablate device (Sonacare Medical) will be described.

